# MiR-126-3p suppresses tumor metastasis and angiogenesis of hepatocellular carcinoma by targeting LRP6 and PIK3R2

**DOI:** 10.1186/s12967-014-0259-1

**Published:** 2014-09-22

**Authors:** Chengli Du, Zhen Lv, Linping Cao, Chaofeng Ding, Owusu-ansah K Gyabaah, Haiyang Xie, Lin Zhou, Jian Wu, Shusen Zheng

**Affiliations:** Department of Hepatobiliary Surgery, The First Affiliated Hospital, Zhejiang University School of Medicine, Hangzhou, 310003 China

**Keywords:** MiR-126-3p, Hepatocellular carcinoma (HCC), Metastasis, Angiogenesis, LRP6, PIK3R2

## Abstract

**Background:**

The deregulation of microRNAs has been reported to play a pivotal role in hepatocellular carcinoma (HCC). MiR-126-3p has been reported to be associated with poor prognosis in HCC. However the underlying mechanism of miR-126-3p in HCC remains unclear.

**Methods:**

The expression levels of miR-126-3p in HCC tissues and cells were detected by RT-PCR. Transwell assay and capillary tube formation assay were applied to assess the metastasis and angiogenesis in vitro. Nude mice subcutaneous tumor model was used to perform in vivo study. Dual- luciferase reporter assay was conducted to confirm the direct binding of miR-126-3p and target genes. The changes of biomarker protein levels were examined by western blot and Immunohistochemistry.

**Results:**

We observed that the miR-126-3p expression levels in HCC tissues and cells were significantly down-regulated. Through gain- and loss- of function studies, we showed that miR-126-3p dramatically inhibited HCC cells from migrating and invading extracellular matrix gel and suppressed capillary tube formation of endothelial cells in vitro. Furthermore, overexpression of miR-126-3p significantly reduced the volume of tumor and microvessel density in vivo. LRP6 and PIK3R2 were identified as targets of miR-126-3p. Silencing LRP6 and PIK3R2 had similar effects of miR-126-3p restoration on metastasis and angiogenesis individually in HCC cells. Furthermore, the miR-126-3p level was inversely correlated with LRP6 and PIK3R2 in HCC tissues. In addition, the rescue experiments indicated that the metastasis and angiogenesis functions of miR-126-3p were mediated by LRP6 and PIK3R2.

**Conclusion:**

Our results demonstrates that deregulation of miR-126-3p contributes to metastasis and angiogenesis in HCC. The restoration of miR-126-3p expression may be a promising strategy for HCC therapy.

**Electronic supplementary material:**

The online version of this article (doi:10.1186/s12967-014-0259-1) contains supplementary material, which is available to authorized users.

## Background

Hepatocellular carcinoma (HCC) is one of the most common human cancers in the world, particularly in China [1]. Active angiogenesis and frequent metastasis are two major characteristics of HCC, which obviously lead to poor prognosis [2]. MicroRNAs (miRNAs) are a class of highly conserved short RNAs that regulate diverse cellular processes by binding to the 3′untranslated region (3′UTR) of target messenger RNAs (mRNAs) [3,4]. Increasing numbers of studies have suggested that miRNAs play an important role in HCC development, such as apoptosis [5,6], proliferation [7,8], autophagy [9], EMT. [10]. To date, relatively few studies have reported that miRNAs possess both metastasis and angiogenesis functions in HCC, such as mir-29b [11], mir-195 [12]. Therefore, miRNAs targeting mRNAs relevant in metastasis and angiogenesis in HCC might be a promising strategy for HCC therapy.

MiR-126-3p has been reported to act as a tumor suppressor by targeting CRK, SOX2, IRS-1 in several cancer types [13-15]. Further studies on zebrafish highlight its significant role in angiogenesis [16,17]. And miR-126-3p has been shown to inhibit angiogenesis in cancer development [18]. The potential role of miR-126-3p in HCC angiogenesis remains unclear. A previous study indicated that miR-126-3p associates with the recurrence rate after liver transplantation and suppresses metastasis in HCC [19]. But the detailed mechanism mediating metastasis remains unknown. In this study, we examined the expression patterns of miR-126-3p in HCC comparing to normal tissue. Both gain and loss of function indicated miR-126-3p suppressed metastasis and angiogenesis in HCC cells. Moreover in vivo study verified the anti-angiogenesis function of miR-126-3p. We then illustrated that LRP6 and PIK3R2 were direct targets of miR-126-3p in mediating anti-metastasis and anti-angiogenesis. Our findings demonstrate the importance of deregulation of miR-126-3p in promoting HCC progression and indicate that miR-126-3p might serves as a therapeutic target for HCC.

## Materials and methods

### Patients and tissue specimens

HCC tissues and their corresponding normal liver tissues were obtained from 70 HCC patients undergoing resection of HCC from 2009 to 2010 in our hospital (First Affiliated Hospital, Zhejiang University School of Medicine, Zhejiang, China). This study was approved by the Ethics Committee of Zhejiang University and informed consent was obtained from each patient.

### Cell lines and culture

Normal liver cell line L02 and liver cancer cell lines HepG2, SMMC-7721, BEL-7402 were purchased from the American Type Culture Collection (Manassas, VA, USA) and the Shanghai Institute of Cell Biology (Shanghai, China). All cell lines were maintained in the recommended culture conditions and incubated at 37°C in a humidified environment containing 5%CO2.

### RNA Oligoribonucleotides construction and lentiviral transduction

MiR-126-3p mimics were purchased from GenePharma (Shanghai, PR China). Anti-miR-126-3p inhibitor was purchased from Invitrogen. Si-LRP-6, si-pik3r2 and its all-star negative control were purchased from Qiagen and their sequences were listed in Additional file 1: Table S1. For lentiviral vector construction, the oligonucleotide of mature miR-126-3p (*5′-*ucguaccgugaguaauaaugcg*-3′*) was chemosynthesized, amplified and cloned into GV209-Puro Vectors by Genechem Co., Ltd (Shanghai, China). The correct sequences and insertions were confirmed by DNA sequencing. For lentiviral infection, cells were plated at a concentration of 3 × 10^4^ cells in 24-well plates overnight and were then infected at MOI of 30 in the presence of polybrene (5 μg/ml) for 8 hours. Infected cells were then cultured for 72 hours with 10% FBS medium. Then puromycin-resistant cell clones were picked and passaged in medium containing puromycin at a concentration of 5 μg/mL. Lentivirus-mediated overexpression of miR-126-3p was verified by qRT-PCR.

### RT-PCR, western blot and Immunohistochemistry (IHC)

The detailed procedure of RNA isolation, RT-PCR, western-blot and immunohistochemistry were performed as described before [20]. GAPDH, RNU6B and β-actin were used as internal control. The primer sequences were listed in Additional file 1: Table S2. The primary antibodies used for western blot and IHC: LRP6 (abcam, ab75358, 1:1000, ab24386, 1:300), beta-catenin (epitomics, 1247–1, 1:1000), PIK3R2 (abcam, ab28356, 1:1000), phospho-ser473-AKT (epitomics, 2118–1, 1:1000), CD34 (epitomics, 2749–1, 1:500), β-actin (A5441, Sigma-Aldrich, 1:2000).

Staining score of CD34, which represented the microvessel density (MVD), was evaluated by counting microvessels (any discrete cluster or single cell). We first scanned the stained microvessels in the 100 × magnification and we chose three most intensely vascularized areasm. Then we counted the microvessels at the magnification of 200× and the mean microvessels number of these three areas were recorded.

Staining score of LRP6 and PIK3R2 was evaluated using a modified Histo-score (H-score), which consisted of a semi-quantitative assessment of both fraction of positive cells (0%-100%) and intensity of staining (no 0, weak 1, moderate 2, or strong 3). The intensity and fraction scores were then multiplied to obtain H-score, which ranged from 0 to 3.

### Isolation and Culture of Human Umbilical Vein Endothelial Cells (HUVECs)

The vein of each umbilical cord was rinsed with 1 × PBS three times, and then perfused and incubated with 0.25% trypsin/0.04% EDTA (Gibico) for 15 min at 37°C with the ends of the vein clamped. Then the trypsin/EDTA solution was centrifuged at 500 g for 5 min and the HUVECs were re-suspended in Serum Free Medium for Endothelial Cells (SFM, Invitrogen), supplemented with 20% FBS, 0.1 mg/ml of heparin and supplements. All HUVECs used in this study were at passages 3–6.

### Preparation of Tumor Cell-Conditioned Medium (TCM)

The cells were plated and transfected in six-well plates. After 48 h, the supernatant medium was removed and replaced by 1.5 ml SFM per well. Following 24 hrs of incubation, the TCM was collected and centrifuged at 5000 g for 10 min to discard cells and cell debris. Then each TCM was adjusted to the number of living cells of corresponding well. The TCM was stored at 80°C until used.

### Capillary tube formation assay

HUVECs (2 × 10^4^/well) were added to matrigel-coated 96-well plates and incubated at 37°C for 6–8 h with 75%TCM. HUVECs were photographed under an inverted microscope. The branch points of the formed tubes, which represent the degree of angiogenesis in vitro, were scanned and quantitated at 100× magnification.

### In vitro assays of migration and invasion

For the migration and invasion assay, 5 × 10^4^ HCC cells were re-suspended in a serum-free medium and placed on the upper chamber of transwell chambers (24-well insert; pore size: 8 μm; BD Biosciences) while culture medium containing 10% FBS was added in the lower chamber. For invasion assay, the membrane was coated with matrigel to form a matrix barrier. The incubation time was 24 hrs for migration and 48 hrs for invasion. The cells on the lower surface of the membrane were fixed in 70% ethanol and stained with crystal violet. Each blue point indicated an individual cell and all the images were scanned and counted at 200× magnification.

### Luciferase reporter assay

Hep-G2 and BEL-7402 cells were seeded in a 24-well plate until 50-70% confluence and then were co-transfected with miR-126-3p or NC and 100 ng of firefly luciferase reporter plasmid that contained either the wild-type or mutant 3′UTR of the target gene. 48 hours after transfection, cells were collected and applied to a dual-luciferase reporter assay (promega, E2920). The tests were repeated in three independent experiments.

### Nude mice xenograft assay

All experimental procedures involving animals were performed in accordance with the Guide for the Care and Use of Laboratory Animals (NIH publications Nos. 80–23, revised 1996), and conforming to the institutional ethical guide-lines for animal experiments. Hep-G2 cells (1 × 10^7^), which were stably expressing mir-126 or NC, were suspended in 100 μl PBS and then injected subcutaneously into the posterior flank of female BALB/c athymic nude mice. Tumor volumes in mice were measured with a slide caliper every week until the scarification. Twelve nude mice were included and they were sacrificed 6 weeks after injection. Results are presented as mean ± standard error of the mean.

### Statistical analysis

The difference between groups was analyzed using a Student t test. The association between mir-126 expression and clinico-pathological variables was assessed by chi-square tests. Statistical analysis was performed with SPSS 15.0 and GraphPad Prism 5.0. P < 0.05 was considered statistically significant.

## Results

### MiR-126-3p is significantly down-regulated in liver cancer tissues and liver cancer cells

**Figure 1:**
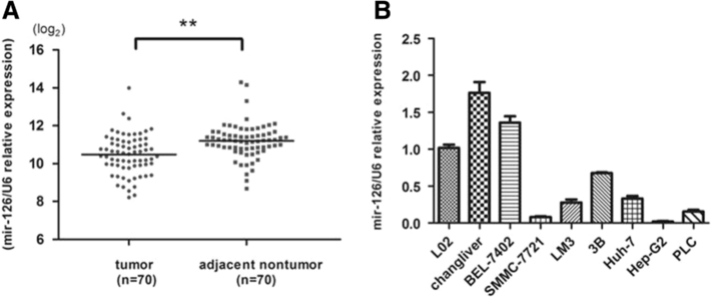
**The expression levels of miR-126-3p in HCC tissues and HCC cell lines. (A)** The level of miR-126-3p in 70 HCC tumors was much lower than corresponding non-tumor tissues. **(B)** Compared to L02, most HCC cell lines displayed much lower expressions of miR-126-3p. Expression levels of miR-126-3p were determined by qRT-PCR and normalized to an endogenous control (U6 RNA). **P < 0.01.

The expression of miR-126-3p was analyzed by RT-PCR and normalized to an endogenous control (U6 RNA). In 70 pairs of HCC tissues, the expression of miR-126-3p was significantly down-regulated in tumors tissues versus adjacent non-tumor liver tissues (Figure 1A). Among these 70 cases, 62 cases revealed a relative lower level in HCC, which suggested that reduction of miR-126-3p was a frequent event in human HCC. To investigate the clinical relevance of miR-126-3p in HCC, the median of all 70 cases was chosen as the cutoff point for separating low-miR-126-3p (n = 35) from high-miR-126-3p expressing tumors (n = 35). Our results showed that miR-126-3p expression was not significantly associated with any single clinicopathological features (Table 1). Then we also measured miR-126-3p expression in a panel of human HCC cell lines as wells as normal liver cell line L02. Similarly, miR-126-3p was down-regulated in most cancer cell lines (Figure 1B). The data above suggests that miR-126-3p decreases in both liver cancer tissues and cells.

**Table 1:** **The relationship between miR-126-3p expression and clinicopathological characteristics in human HCC patients**

**Variables**	**All patients**	**miR-126-3p expression**		***P*** **value**
	**(n = 70)**	Low*	High*	
**Age (years)**				
**≤55**	24	12	12	1
**>55**	46	23	23	
**Gender**				
**Male**	62	31	31	1
**Female**	8	4	4	
**Size of tumor (cm)**				
**≤5**	30	12	18	0.147
**>5**	40	23	17	
**Number of tumor**				
**Single**	54	25	29	0.255
**Multiple**	16	10	6	
**Portal vein invasion**				
**Negative**	46	22	24	0.615
**Positive**	24	13	11	
**TNM staging**				
**I, II**	37	15	23	0.094
**III**	33	20	13	
**Grade**				
**Well + Moderate**	61	29	32	0.284
**Poor**	9	6	3	
**AFP (ng/ml)**				
**≤400**	39	16	23	0.092
**>400**	31	19	12	

*The median expression level was used as the cut-off. Low expression of miR-126-3p in 35 patients was classified as values below the 50th percentile. High miR-126-3p expression in 35 patients was classified as values at or above the 50th percentile.

For analysis of correlation between miR-126-3p expressions and clinical features, chi-square tests were used. Results were considered statistically significant at P <0.05.

### MiR-126-3p inhibits tumor metastasis and angiogenesis in vitro

**Figure 2:**
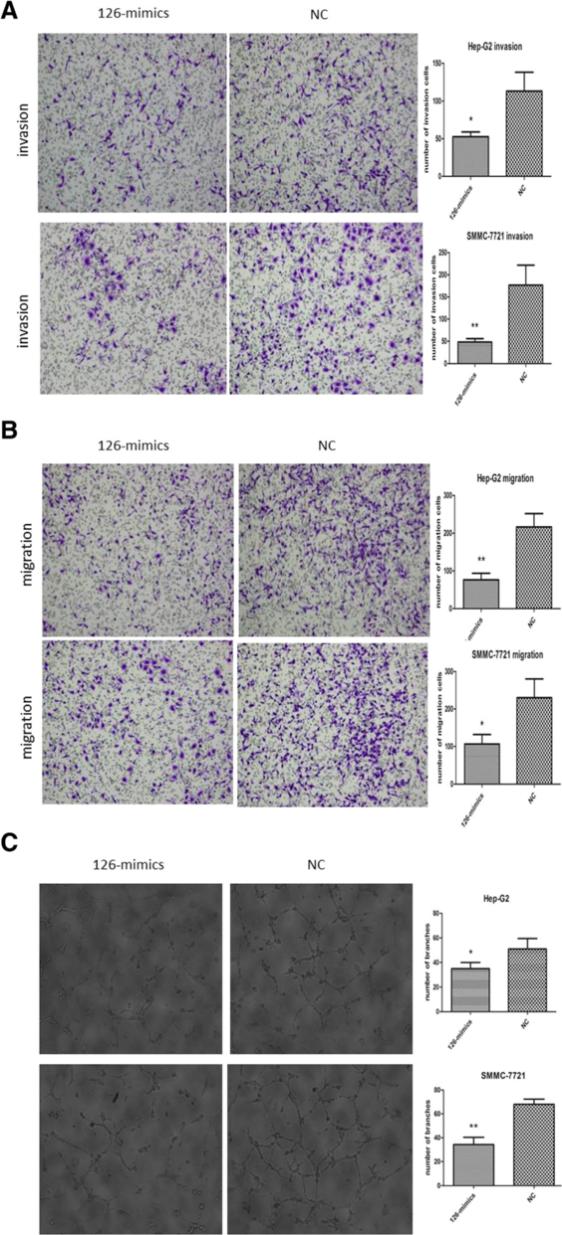
**MiR-126-3p inhibits tumor metastasis and angiogenesis in vitro. (A, B)** Representative images of invasion **(A)** and migration **(B)** assays of Hep-G2 cells and SMMC-7721 cells transfected with miR-126-3p mimics or NC. **(C)** HUVECs were cultured in the presence of 75% TCM from Hep-G2 and SMMC-7721 cells transfected with miR-126-3p mimics or NC. Representative images of tube formation assays and the number of branch points were shown. NC indicated negative control. *P < 0.05. **P < 0.01.

To explore the potential role of miR-126-3p in HCC metastasis, we transfected HCC cells with miR-126-3p mimics (Additional file 2: Figure S1C) and performed in vitro transwell assays in HepG2 and SMMC-7721 cells, which displayed lowest expressions of miR-126-3p. Compared to NC, HCC cells transfected with miR-126-3p mimics significantly reduced the number of cells invading or migrating the chamber membrane with or without matrigel (Figure 2A,B).

We next performed capillary tube formation assay to elucidate the role of miR-126-3p in HCC angiogenesis. TCM from HepG2 or SMMC-7721 cells transfected with miR-126-3p mimics inhibited HUVECs to form capillary-like structures compared with TCM from cells transfected with NC (Figure 2C).

Then we carried out loss-of function study by using miR-126-3p inhibitor, which dramatically decreased the endogenous level of miR-126-3p to further verify the function of miR-126-3p in HCC (Additional file 2: Figure S1C). The HCC cell line we selected was BEL-7402, which expressed relatively high level of miR-126-3p. As expected, suppression of miR-126-3p in BEL-7402 promoted the ability of metastasis and angiogenesis (Additional file 3: Figure S2A,B). Collectively, both gain- of function (GOF) and loss- of function (LOF) studies suggest the suppressive effects of miR-126-3p on HCC cells metastasis and angiogenesis in vitro.

### MiR-126-3p inhibits tumor proliferation and angiogenesis in vivo

**Figure 3:**
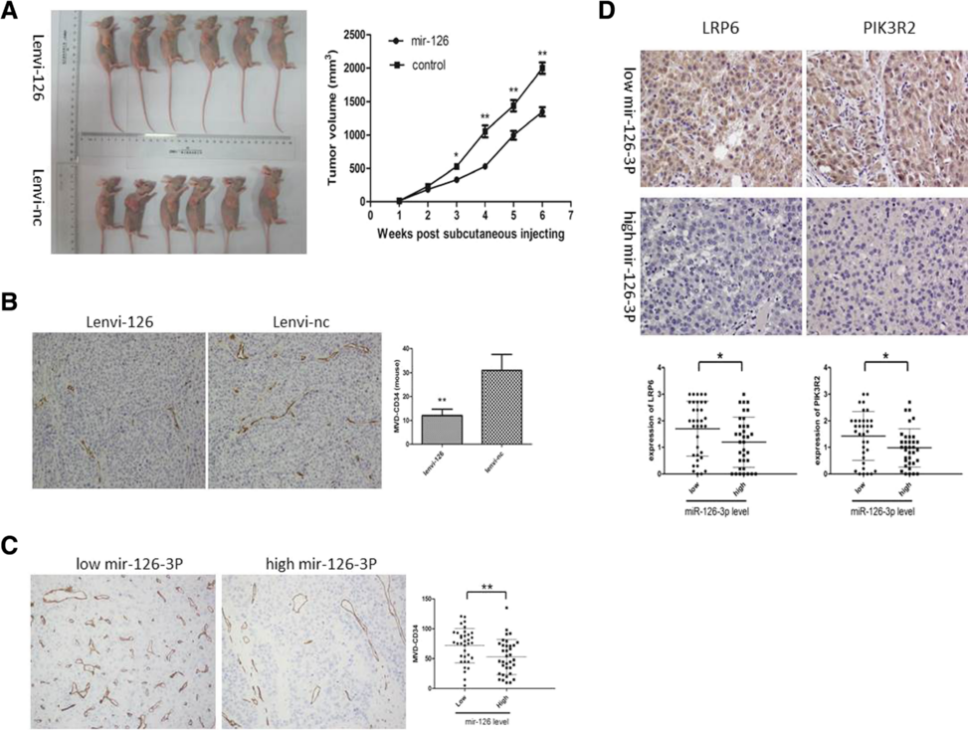
**Mir-126-3p suppresses tumor growth and angiogenesis in vivo. (A)** Overexpression of miR-126-3p significantly suppressed the growth of subcutaneous tumor growth. Growth curve and subcutaneous xenograft tumors removed were shown. **(B)** Subcutaneous xenograft tumors of lenvi-miR-126 or lenvi-NC were immunohistochemically stained for CD34. Tumors of lenvi-miR-126 displayed lower microvessel density. **(C, D)** HCC with lower miR-126-3p expression showed higher MVD, LRP6 and PIK3R2. MVD was evaluated based on CD34 staining and was determined from the three most intensely vascularized areas of each section at a magnification of 200×. The evaluation of LRP6 and PIK3R2 was performed at a magnification of 400×. The median of all 70 cases was chosen as the cutoff point for separating low miR-126-3p group (n = 35) from high miR-126-3p group (n = 35) in **(C)**. *P < 0.05. **P < 0.01.

To further validate the role of miR-126-3p in HCC, we performed in vivo assays by subcutaneously injecting Hep-G2 cells stably expressing miR-126-3p or control vector (Additional file 2: Figure S1B). The subcutaneous tumors were observed and measured regularly, and mice were sacrificed 6 weeks after HCC cells implantation. We found that the average tumor volume of Hep-G2 cells stably overexpressing miR-126-3p was significantly smaller than their control group (Figure 3A).

Next, we excised the subcutaneous tumors and performed IHC to detect the expression level of CD34 of each group, which indicates the micro-vessels expression. As shown in Figure 3B, the group of miR-126-3p overexpression displayed dramatically lower level of CD34, compared to the group of NC. Then, we further analyzed the association between miR-126-3p level and angiogenesis in the same 70 human HCC tissues. Obviously, the MVD level was inversely correlated with miR-126-3p expression (Figure 3C). The results above indicate that miR-126-3p suppresses tumor proliferation and angiogenesis in vivo.

### MiR-126-3p inhibits HCC metastasis by directly targeting LRP6

**Figure 4:**
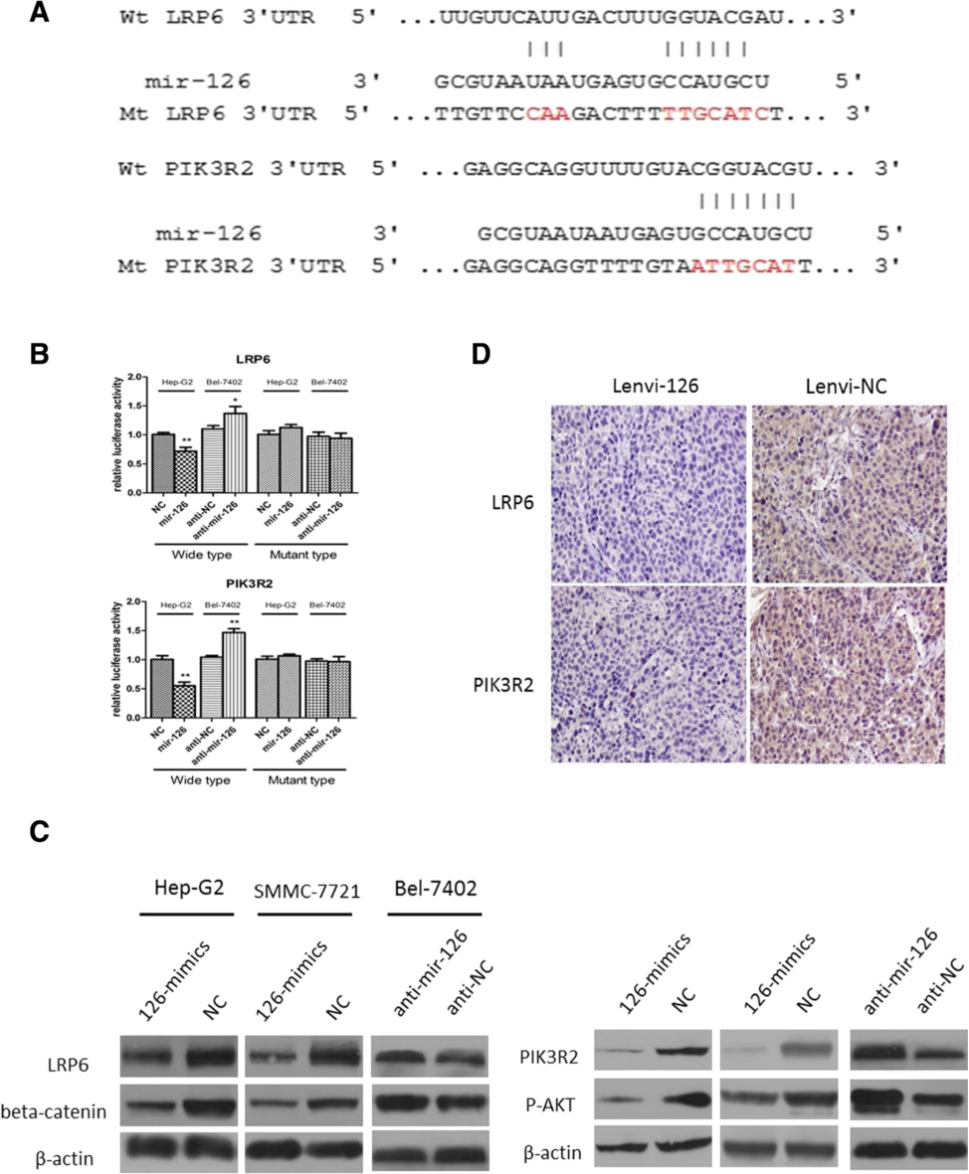
**LRP6 and PIK3R2 are direct targets of miR-126-3p. (A)** MiR-126-3p and its putative binding sequence in the 3′UTR of LRP6 and PIK3R2. The mutant miR-126-3p binding site was generated in the complementary site for the seed region of miR-126-3p. **(B)** Hep-G2 and BEL-7402 cells were co-transfected with miR-126-3p mimics or NC and wild-type or its mutant-type (either LRP6 or PIK3R2), and the luciferase activities were examined. The firefly luciferase activity of each sample was normalized to the Renilla luciferase activity. **(C)** Western blot results of endogenous LRP6, PIK3R2, beta-catenin and p-AKT proteins in HCC cells transfected with miR-126-3p mimics or anti-mir-126-3p and its NC. **(D)** Analysis of LRP6 and PIK3R2 expression in subcutaneous tumors by immunohistochemistry. NC indicated negative control, **P < 0.01.

Next, we searched for a candidate target gene responsible for metastasis suppression of miR-126-3p in HCC cells. By comprehensive analysis of miRanda, TargetScan, and PicTar algorithms, LRP6 was chosen as a candidate target of mir-126 (Figure 4A), which had been reported to have a suppressive role of metastasis in HCC and reported as a target in 293 T cells [21]. The dual-luciferase reporter assays in Hep-G2 cells and BEL-7402 cells revealed that co-transfection of miR-126-3p mimics significantly inhibited the activity of firefly luciferase reporter with wild-type 3′UTR of LRP6 but not mutant 3′UTR of LRP6, while the inhibition of miR-126-3p revealed an opposite result (Figure 4B). Further, western blot assay indicated that the expression level of miR-126-3p inversely correlated with LRP6 as well as its downstream target beta-catenin (Figure 4C). Moreover, IHC of xenografts from the miR-126-3p group showed dramatically lower expression of LRP6 compared to the NC group (Figure 4D). In addition, in these 70 HCC cases, miR-126-3p level was inversely correlated with LRP6 expression (Figure 3D).

**Figure 5:**
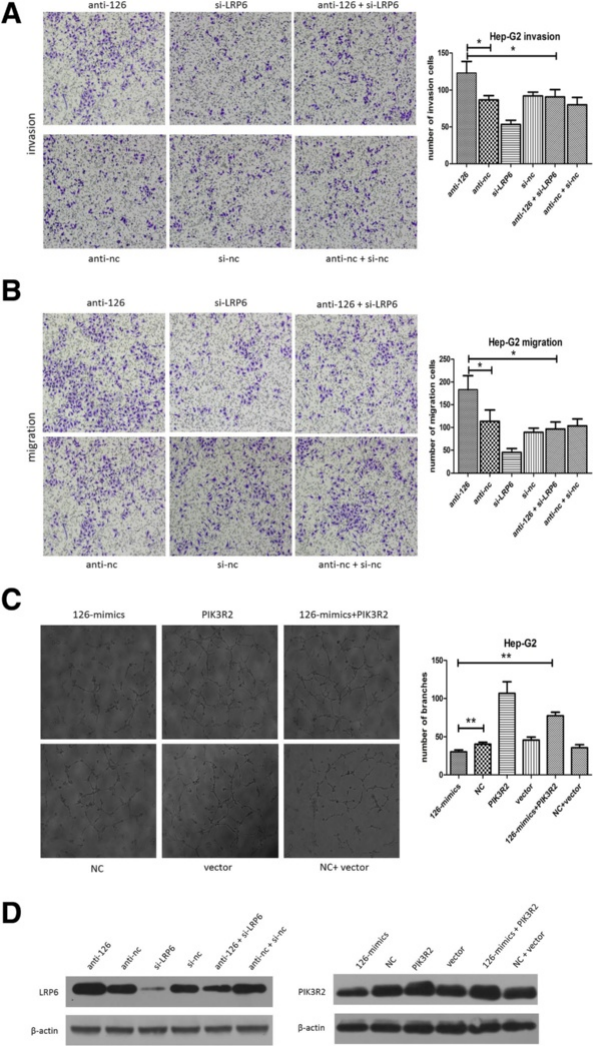
**The effects of LRP6 and PIK3R2 alterations on miR-126-3p functions in Hep-G2 cells. (A, B)** Both invasion and migration assays showed that the trans-cells of the “rescue” group (anti-126 + si-LRP6) significantly reduced compared with anti-126 group **(C)** Tube formation assays showed that the group co-transfected with PIK3R2 and 126-mimics formed more tube branches than 126-mimics group **(D)** Western bolt was performed to assess the protein levels of LRP6 and PIK3R2 in co-transfection assays. NC indicated negative control, *P < 0.05. **P < 0.01.

Then, we explored the role of LRP6 in HCC metastasis. As is shown in Additional file 4: Figure S3A, si-LRP6 obviously decreased the ability of migration and invasion of HCC cells. To verify the vital role of LRP6 in mediating miR-126-3p’s effects on metastasis, BEL-7402 cells were co-transfected with anti-miR-126-3p and si-LRP6. As a result, si-LRP6 suppression attenuated the pro-metastasis effects of anti-mir-126 (Figure 5A,B). We then explored the protein level of LRP6 in each group, which was proved to conform to the functional outcomes (Figure 5D). These results provided strong evidence that LRP6 is a target of miR-126-3p in HCC and mediates the metastasis function of miR-126-3p in HCC.

### MiR-126-3p repressed HCC angiogenesis by negatively regulating PIK3R2/P-AKT expression

As both in vitro and in vitro assays showed that miR-126-3p inhibited angiogenesis, we then explored the molecular mechanism underlying the effects. Among the predicted targets of miR-126-3p, we chose PIK3R2 as the potential target mediating angiogenesis of mir-126, which is well known to phosphorylate AKT and then promoted angiogenesis [22,23]. Like reported in other researches in 293 T cells [16,17], we demonstrated that miR-126-3p was shown to directly bind to the 3′UTR of PIK3R2 by both up-regulation and inhibition of miR-126-3p in Hep-G2 and BEL-7402 cells (Figure 4B). While we enhanced the expression level of miR-126-3p in HCC cells, western blot displayed a decrease protein level of PIK3R2 and phosphorylation of AKT. Further the inhibition of miR-126-3p enhanced the expression of PIK3R2 and P-AKT (Figure 4C). Besides, 70 HCC tissues and subcutaneous tumors from mice also indicated an inverse correlation of miR-126-3p and PIK3R2 (Figures 3C, 4D).

Then we suppressed the level of PIK3R2 in HCC cells to elucidate its role in HCC angiogenesis. Similar to miR-126-3p overexpression, silencing of PIK3R2 impaired the formation of capillary-like structures of HUVECs (Additional file 4: Figure S3B). We further performed “rescue” study in Hep-G2 cells, namely, TCM from co-transfection of miR-126-3p mimics and PIK3R2-ORF partially attenuated the anti-angiogenesis effects of TCM from miR-126-3p mimics (Figure 5C). Additionally, the PIK3R2 level showed a similar result in the “rescue” assay (Figure 5D). These data suggests that miR-126-3p suppresses angiogenesis in HCC through PIK3R2/P-AKT pathway.

## Discussion

It is well known that HCC is a malignant cancer characterized by frequent metastasis and angiogenesis. To date, mRNAs and microRNAs associated with HCC development are innumerably reported, among which mRNAs and microRNAs pro- or anti- metastasis and angiogenesis remain highlights in recent several years, such as twist1 [24], CXCR6 [25], vasohibin2 [26], mir-29b [6], mir-195 [12]. And miRNAs possessing anti- metastasis and angiogenesis functions are thought to be a novel anti-HCC targets.

In a previous study, miR-126-3p has been reported to be associated with poor survival after liver transplantation by promoting metastasis in HCC [19]. Here, we, for the first time, illuminate down-regulation of miR-126-3p expression in HCC. And both gain- and loss- of function assays indicated that miR-126-3p plays a vital role in anti-metastasis and anti-angiogenesis in vitro. Further in vivo assay, miR-126-3p was inversely correlated with vessel marker CD34. And tumor sizes of miR-126-3p overexpression group were much smaller, which might be attributed to less microvessels. Moreover, in the same 70 HCC tissues, the expressions of miR-126-3p and CD34 also revealed an inverse correlation.

The fundamental way mediating functions of miRNAs is to regulate their target genes by direct cleavage of the mRNA or by inhibition of protein synthesis [27]. We then disclosed that miR-126-3p inhibited metastasis and angiogenesis by targeting LRP6 and PIK3R2, respectively. And the evidence is listed as follows: 1) the protein levels of LRP6 and PIK3R2 were decreased after overexpression of miR-126-3p in vitro or in vivo. 2) Overexpression of miR-126-3p decreased the luciferase reporter activity of wild-type 3′UTR but not mutant 3′UTR of LRP6 and PIK3R2 and inhibition of miR-126-3p possessed the opposite results. 3) The effects of miR-126-3p overexpression on metastasis and angiogenesis could be phenocopied by suppression of LRP6 and PIK3R2. And co-transfected of si-LRP6 or restoration of PIK3R2 partly abrogated the effects induced by anti-miR-126-3p or miR-126-3p mimics, respectively. 4) miR-126-3p level was inversely correlated with LRP6 and PIK3R2 in HCC tissues.

LRP6 is a well-known activator of beta-catenin, leading to pro-proliferation, pro-metastasis and anti-apoptosis in several cancer types including HCC [21,28,29]. Here, we demonstrated that overexpression of miR-126-3p led to down-regulation of LRP6 and its downstream beta-catenin in vitro and in vivo studies. As LRP6/beta-catenin pathway is frequently activated in HCC [21,30], the down-regulation of miR-126-3p might account for this phenomenon. Additionally, PIK3R2, which has been reported to be a target of miR-126-3p [16,31], was verified to be inversely correlated with miR-126-3p. We illuminated that overexpression of miR-126-3p significantly inhibited PIK3R2/P-AKT pathway in HCC cells and xenografts. PIK3R2/P-AKT pathway, which is closely related to angiogenesis [22,23], mediates the effects of miR-126-3p anti-angiogenesis. Recently, PI3K/P-AKT pathway has been demonstrated to play a vital role in HCC development [32,33]. PI3K/P-AKT inhibitors, such as GSK690693, AZD5363 [34,35], are shown to inhibit the phosphorylation of AKT and then suppress the progress of cancers. Therefore, the new miR-126-3p /PIK3R2/P-AKT pathway might serve as a promising therapeutic target, at least in terms of angiogenesis.

In conclusion, our results show that miR-126-3p is down-regulated in HCC. Both gain- of function and loss- of function assays indicate that miR-126-3p suppresses metastasis and angiogenesis in HCC cells. We then investigate the underlying mechanism and find that miR-126-3p possesses the effects by direct targeting LRP6 and PIK3R2. So, restoration of miR-126-3p may represent a promising strategy for anti-HCC therapy.

## Conclusion

In this study, we demonstrate that the down-regulation of miR-126-3p promotes metastasis and angiogenesis by targeting LRP6 and PIK3R2 in HCC. So restoration of miR-126-3p might be a candidate therapeutic target for HCC patients.

## Additional files

Additional file 1: Table S1 The sequences of siRNAs. **Table S2.** Primers used in this study. Additional file 2: Figure S1 RNA oligoribonucleotide and lentiviral transduction effects on miR-126-3p expression in HCC cells. Additional file 3: Figure S2 Loss of function assays of miR-126-3p effects on metastasis and angiogenesis in vitro. Additional file 4: Figure S3 Suppression of LRP6 and PIK3R2 inhibits metastasis and angiogenesis in vitro respectively.
