# Supplementary material for: MiR-126-3p suppresses tumor metastasis and angiogenesis of hepatocellular carcinoma by targeting LRP6 and PIK3R2
Source: J Transl Med. 2014 Sep 22;12:259. doi: 10.1186/s12967-014-0259-1 (PMC4189615; doi:10.1186/s12967-014-0259-1)
Supplement: Additional file 1: Table S1 — The sequences of siRNAs. Table S2. Primers used in this study. [file 12967_2014_259_MOESM1_ESM.doc]

**Supporting Table 1. siRNA sequence**

| **Name** | **sequence** |
| --- | --- |
| si-LRP6 | CTGGATGGTTCTGACCGTGTA |
| si-PIK3R2 | CAGGGCCAGACTCAAGAGAAA |

**Supporting Table 2.** Primers for RT-PCR

| **Name** | **Sense Strand (5' - 3')** | **Antisense Strand (5' - 3')** |
| --- | --- | --- |
| GAPDH | GTCTCCTCTGACTTCAACAGCG | ACCACCCTGTTGCTGTAGCCAA |
| LRP6 | CAGTTGGAGTGGTGCTGAAAGG | CCATCCAAAGCAGCCCGTTCAA |
| PIK3R2 | ATGGCACCTTCCTAGTCCGAGA | CTCTGAGAAGCCATAGTGCCCA |
