# Supplementary figures and images for: MiR-126-3p suppresses tumor metastasis and angiogenesis of hepatocellular carcinoma by targeting LRP6 and PIK3R2
Source: J Transl Med. 2014 Sep 22;12:259. doi: 10.1186/s12967-014-0259-1 (PMC4189615; doi:10.1186/s12967-014-0259-1)

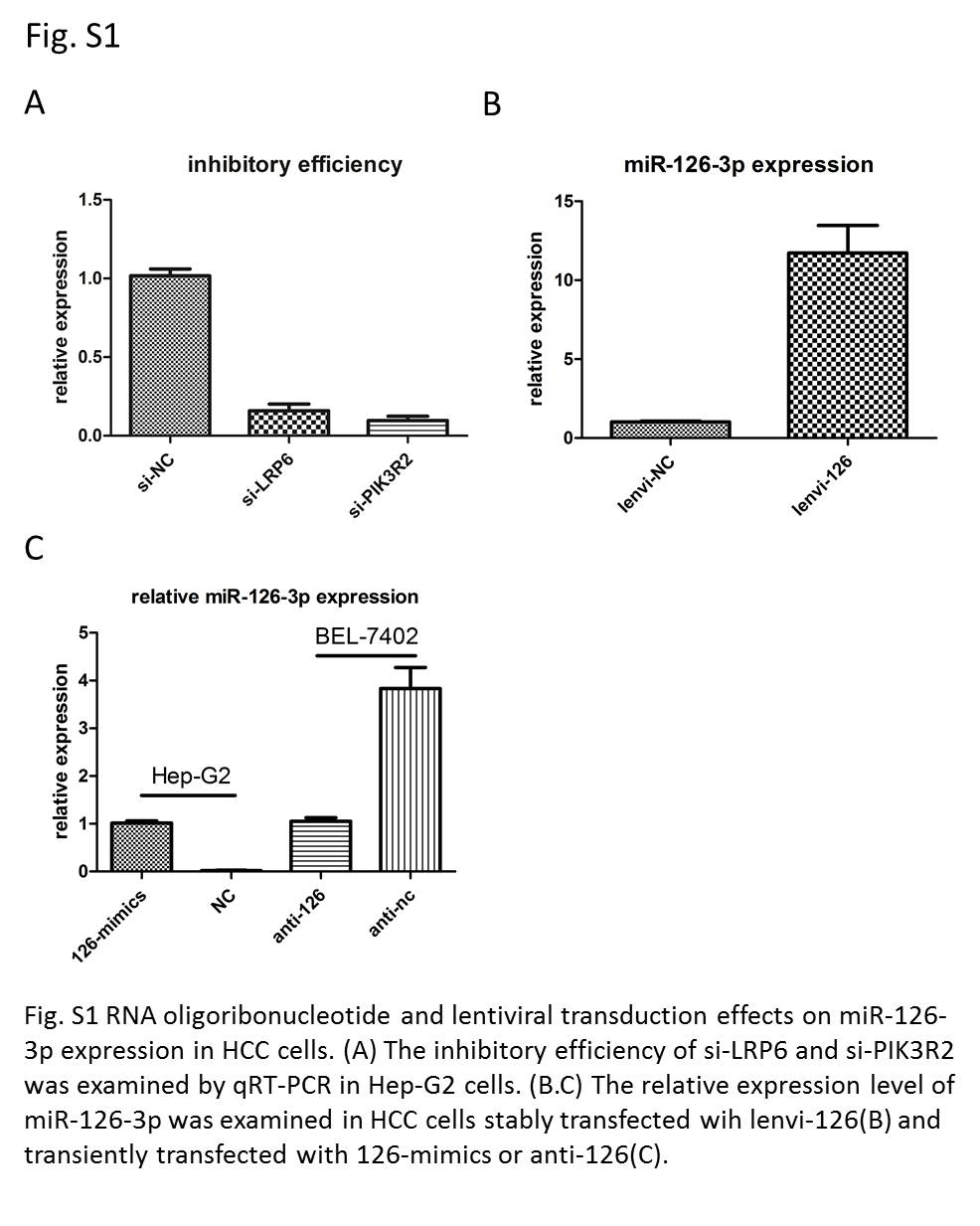

Supplement: Additional file 2: Figure S1 — RNA oligoribonucleotide and lentiviral transduction effects on miR-126-3p expression in HCC cells. [file 12967_2014_259_MOESM2_ESM.jpeg]

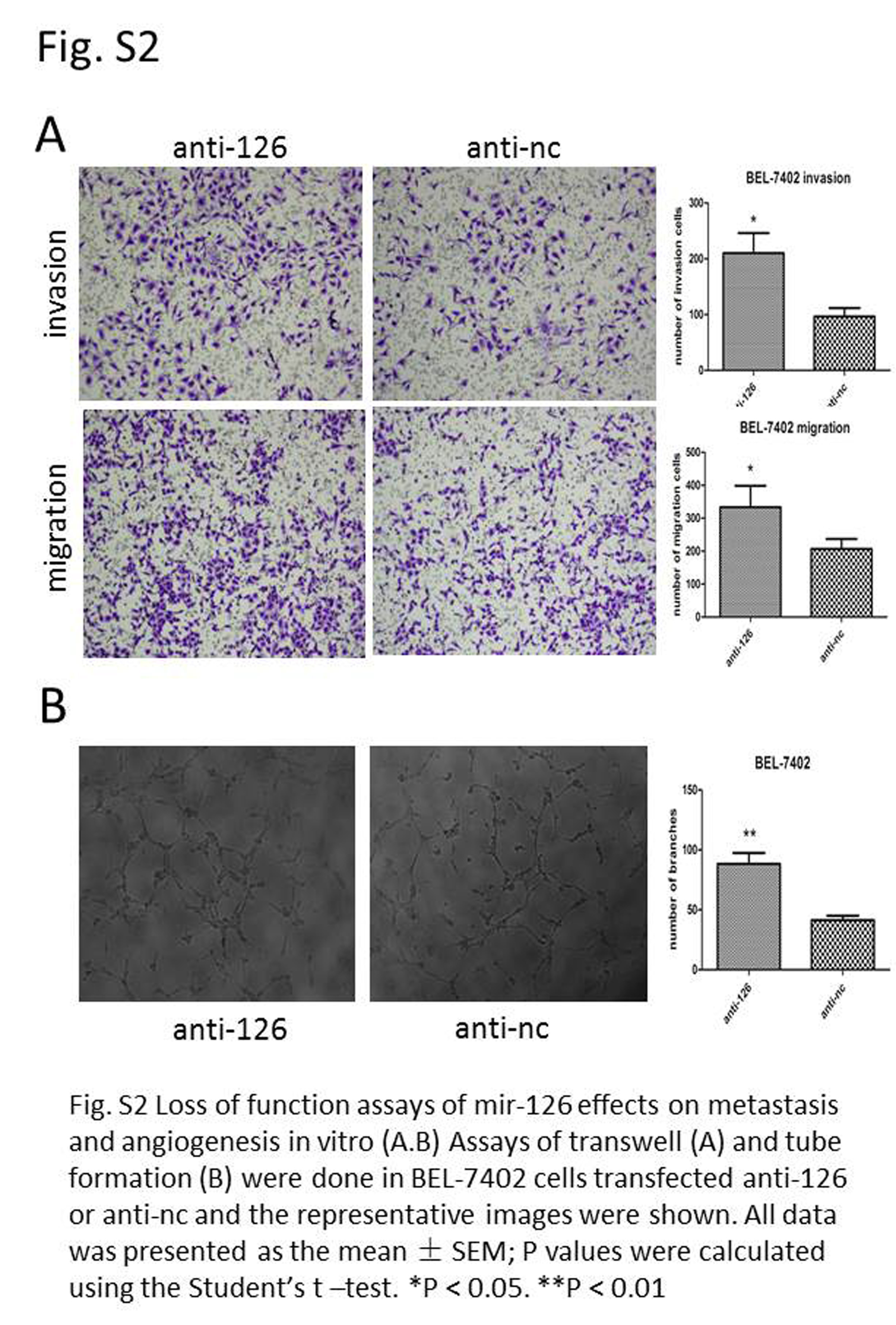

Supplement: Additional file 3: Figure S2 — Loss of function assays of miR-126-3p effects on metastasis and angiogenesis in vitro. [file 12967_2014_259_MOESM3_ESM.jpeg]

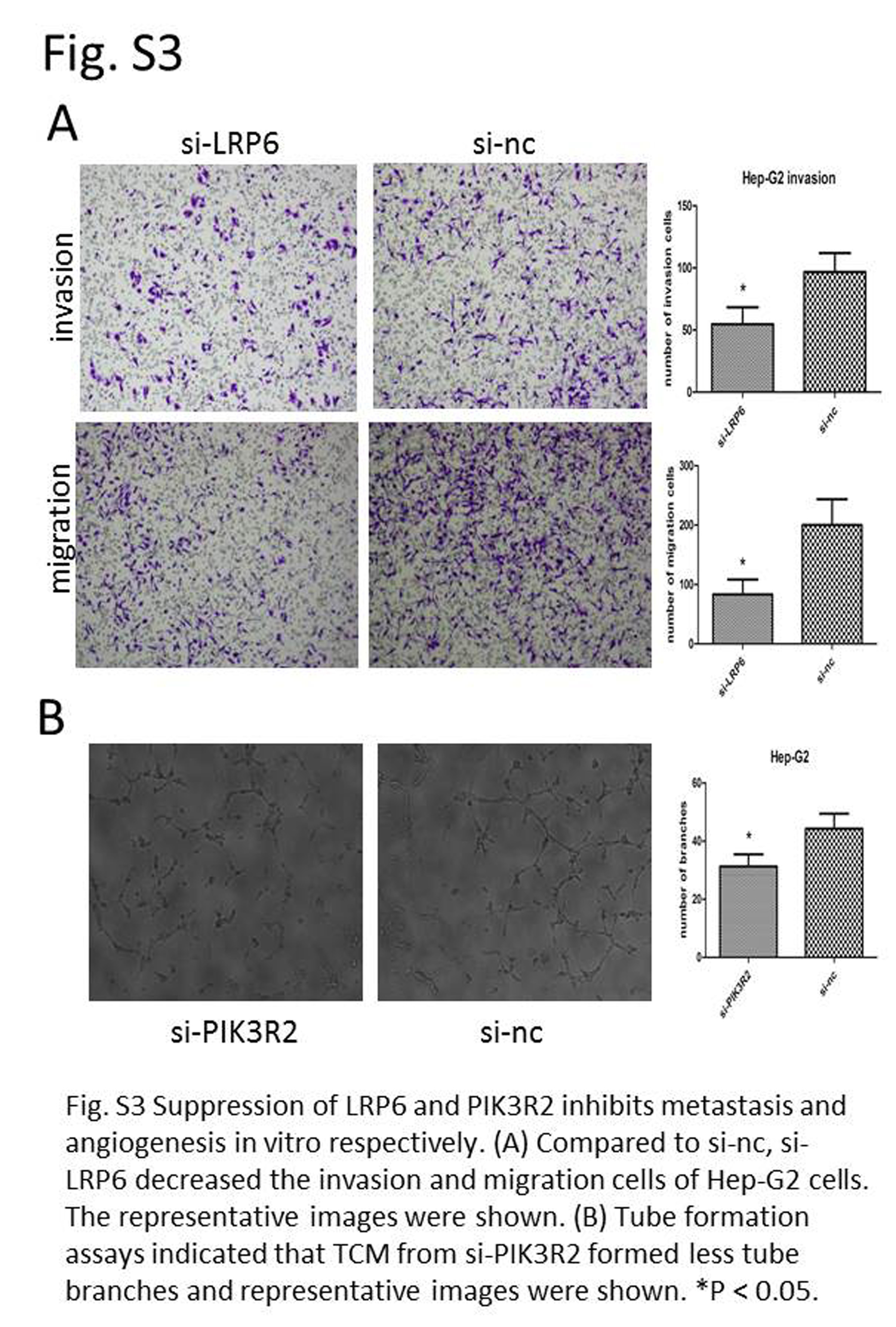

Supplement: Additional file 4: Figure S3 — Suppression of LRP6 and PIK3R2 inhibits metastasis and angiogenesis in vitro respectively. [file 12967_2014_259_MOESM4_ESM.jpeg]
